# Three-dimensional virtual planning of corrective osteotomies of distal radius malunions: a systematic review and meta-analysis

**DOI:** 10.1007/s11751-017-0284-8

**Published:** 2017-04-25

**Authors:** R. J. O. de Muinck Keizer, K. M. Lechner, M. A. M. Mulders, N. W. L. Schep, D. Eygendaal, J. C. Goslings

**Affiliations:** 1 Trauma Unit, G4-137, Department of Surgery, Academic Medical Center, PO-box 22660, 1100 DD Amsterdam, The Netherlands; 2 Department of Surgery, Maasstad Hospital, Rotterdam, The Netherlands; 3 Department of Orthopaedic Surgery, Amphia Hospital, Breda, The Netherlands

**Keywords:** 3-Dimensional, Corrective osteotomy, Distal radius, Malunion

## Abstract

The purpose of this study was to summarize and evaluate results of three-dimensional (3D-) planned corrective osteotomies of malunited distal radius fractures. 3D-planning techniques provide the possibility to address 3D-deformity that conventional planning methods might not address. We systematically searched PubMed, EMBASE and the Cochrane library for studies that performed a 3D-planned corrective osteotomy on patients with a malunited distal radius fracture. Fifteen studies with a total of 68 patients were included in the analysis. In 96% of cases, the preoperatively present palmar tilt, radial inclination and ulnar variance showed statistically significant improvement postoperatively with restoration to within 5° or 2 mm of their normal values. Mean flexion–extension, pro-supination and grip strength showed statistically significant improvement (*p* < 0.05). Complications were reported in 11 out of 68 patients (16%). With the current advances in 3D printing technology, 3D-planned corrective osteotomies seem a promising technique in the treatment of complex distal radius malunions.

*Level of evidence IV* Systematic review of case series, Level IV.

## Introduction

Malunion of distal radius fractures is a frequently seen complication, occurring in approximately 5% of distal radius fractures [1]. Up to 83% of malunited distal radius fractures are symptomatic, causing pain, weakness or functional impairment of the joint [1–3]. These symptomatic malunited distal radius fractures often require surgical correction to restore the anatomy of the wrist and improve functional outcome.

The indication for surgical correction is predominantly based on the degree of functional impairment and correctable radiographic findings that potentially cause the patients’ complaints [3, 4]. The functional impact of the deformity is patient-specific, depending on the age, dominance of the affected arm and activity level of the patient [3, 5].

Acceptable limits of radiographic deformation have been established for the distal radius (Table 1) [3, 6, 7]. Within these limits, symptoms of distal radius malunions are expected to be minimal [8]. Nonetheless, acceptable values vary between individuals. Often the unaffected contralateral forearm of the patient is used as a reference to evaluate patient-specific degrees of malformation [9–11].

**Table 1:** Radiographic evaluation of the distal radius; normal values and acceptable limits of deformity [3, 6, 7]

Parameter	Normal value	Acceptable limit of deformity
Radial inclination	21–25°	>15°
Radial length or height	10–13 mm	7–15 mm
Ulnar variance	Neutral, ±1 mm	<3 mm compared to contralateral side
Dorsal–volar angulation	11° volar	≤15° dorsal tilt, ≤20° volar tilt

Several studies have shown that accurate anatomic reconstruction of the malunited radius can improve functional outcome in patients with a symptomatic malunion [11–13]. A corrective osteotomy is the treatment of choice to restore the anatomic configuration and optimize functional outcome [5, 10, 11, 14].

In order to optimize accuracy of the planned corrective osteotomy, extensive preoperative planning is indispensable. Radiographic evaluation of the affected limb aids in obtaining details of the deformity and determining the osteotomy plane, the fixation method, and in some cases the shaping of a bone graft [8, 15, 16].

Traditionally, preoperative planning is based on 2 orthogonal radiographs depicting lateral and posteroanterior views of the radius [11, 17, 18]. With this method, however, complex deformations are often not addressed [18–20]. Especially, rotational deformities are difficult to assess on plain radiographs [8, 15, 18]. Computer-assisted techniques with three-dimensional (3-D) images and models address 3-D deformity and may further optimize functional and radiographic results of corrective osteotomies [21–24].

3D-planned corrective osteotomies usually involve three steps [19, 25]. Firstly, data are collected by obtaining a CAT scan of the malunited and contralateral healthy forearm. Secondly, virtual models are created of both radii. By superimposing the malunited radius on a mirrored version of the healthy contralateral side, the location and degree of deformity is determined. Subsequently, a virtual cutting plane is set within the region of the malunion, which divides the bone in a proximal and distal part. The distal and proximal part of the malunited radius can be rotated and translated to match with the contralateral radius [26]. With the third and last step, the preoperative plan is translated to the patient during actual surgery [21, 22].

Transferal of the planned osteotomy to the patient’s bone is a delicate task for which multiple solutions have been suggested. In its simplest form, virtual or physical 3D models aid the surgeon in understanding and visualizing the planned osteotomy plane [27]. Additionally, there is the possibility to guide the reposition with optical tracking devices [19, 28]. Another option is the use of synthetic templates that can be placed in the osteotomy gap, thereby restoring the original position of the distal radius [23, 29]. Ultimately, 3D-planning techniques provide the possibility to create patient-specific surgical cutting guides and fixation plates [21, 22, 26, 27, 30–34]. Templates are made to match the patients’ anatomy and include drilling guides and one or more osteotomy slits. Successively, the corrected position can be secured with the use of preoperatively defined, patient-specific plates.

Advances in computer technology and 3D printing facilities have made these techniques more accessible in daily clinical practice [35]. Therefore, the aim of this study was to assess the results of corrective osteotomies of a malunited distal radius with the use of 3D planning techniques by systematically evaluating the available literature.

## Methods

This systematic review was performed in accordance with the PRISMA checklist for systematic reviews [36].

### Search strategy and inclusion criteria

In collaboration with a clinical librarian, two authors (RJODMK and KML) jointly performed a search of the medical databases MEDLINE, EMBASE and the Cochrane Central Register of Controlled trials. The search strategy was used for PubMed and adapted for each database (Table 2). All English, German and Dutch titles published between January 1, 2000, and February 1, 2016, were considered. We included systematic reviews, randomized controlled trials, cohort studies, case series and case reports. Only studies describing patients with a posttraumatic malunion of the distal radius were included. Deformities due to growth disturbances or congenital anomalies were not considered, nor were diaphyseal or bilateral malunions. Studies applying a 3D-planned corrective osteotomy solely on phantoms or cadavers were excluded, as were descriptive technical reports that did not perform a 3D-planned corrective osteotomy. The osteotomy was considered as ‘3D-planned’ if the preoperative planning was based on computer-assisted three-dimensional images of both the malunited and uninjured distal radius.

**Table 2:** PubMed search

Strategy	#1 AND #2 AND #3
#1	“Colles’ Fracture”[Mesh] OR colles’ fracture*[tiab] OR colles fracture*[tiab] OR radius fracture[Mesh] OR distal radius fracture*[tiab] OR radius[tiab] OR distal radial[tiab]
#2	Three dimensional[tiab] OR 3d[tiab] OR 3-D[tiab] OR computer assisted[tiab] OR computer-assisted [tiab] OR computer simulation[tiab] OR patient specific instrument[tiab] OR virtual planning[tiab] OR computer aided[tiab] OR computer-aided[tiab]
#3	((“Fractures, Malunited”[Mesh] OR malunited fracture*[tiab] OR malunion[tiab] OR cross united fracture*[tiab] OR abnormal union fracture*[tiab] OR deformity[tiab])) OR (“Osteotomy”[Mesh] OR osteotomy[tiab] OR osteotomies[tiab])

All records from the electronic search were screened on title and abstract by two authors (RJODMK and KML). Disagreement was resolved by the consultation of a third reviewer. Of the selected articles, full texts were assessed for eligibility. Subsequently, the reference list of all included studies was screened for potentially relevant studies.

### Outcome measures

The primary outcome measure was the functional outcome including range of motion (ROM) of the wrist and/or forearm and/or grip strength. Range of motion comprised flexion and extension and pro- and supination. Our secondary outcomes were radiological outcome, including palmar tilt, radial inclination, ulnar variance and rotational angle, and complications.

### Quality assessment

To determine the quality of the included studies, we used the checklist suggested by the Delphi panel for case series [37]. This checklist consists of six main topics subdivided in 17 criteria (“Appendix” section). The 17 criteria were scored on how well these were described: 3 points were allocated if it was clearly defined, 1 point if it was partially or inadequately defined, and 0 points if it was not defined. Subsequently, subscores were calculated per main topic and labeled with a color depending on its score, respectively, green (good), orange (medium) or red (not described). The points needed for a specific color are shown in Table 3. A study was considered as ‘high quality’ if four or more topics were scored with a green label, ‘low quality’ if three or more topics were scored with a red label and ‘medium quality’ for all other combinations.

**Table 3:**
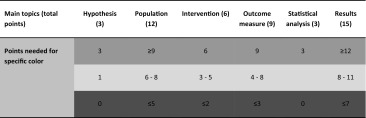
Scoring scheme for quality assessment (color figure online)

### Data collection and statistical analysis

The data of the individual articles were extracted by one author (KML) on a pre-piloted data extraction form. All data on patient characteristics, used technique, functional and radiographic results and complications were extracted. Additionally, we performed an individual patient data meta-analysis (IPDMA) in order to produce a more precise overall estimate of the average effect [38]. To optimize quality of the IPDMA, authors of included studies were contacted to provide additional data on age of patients, time between the fracture and the correction of the malunion, time until bony union and both pre- and postoperative functional and radiographic parameters. To facilitate IPD analyses, bi-directional range of motion was transposed into a single range (e.g., flexion 40°, extension 25°: flexion–extension range of 65°). Radiographic measurements on pre- and postoperative palmar tilt, radial inclination and ulnar variance were transposed to their distance to normal values (11° palmar tilt, 23° radial inclination and neutral (0 mm) ulnar variance, respectively).

Means and standard deviations were calculated for the available data. In case of normal distribution, we used a paired *T* test to check for statistical significant improvement. For non-normally distributed data, a Wilcoxon signed rank test was used.

## Results

### Literature search and quality assessment

**Fig. 1:**
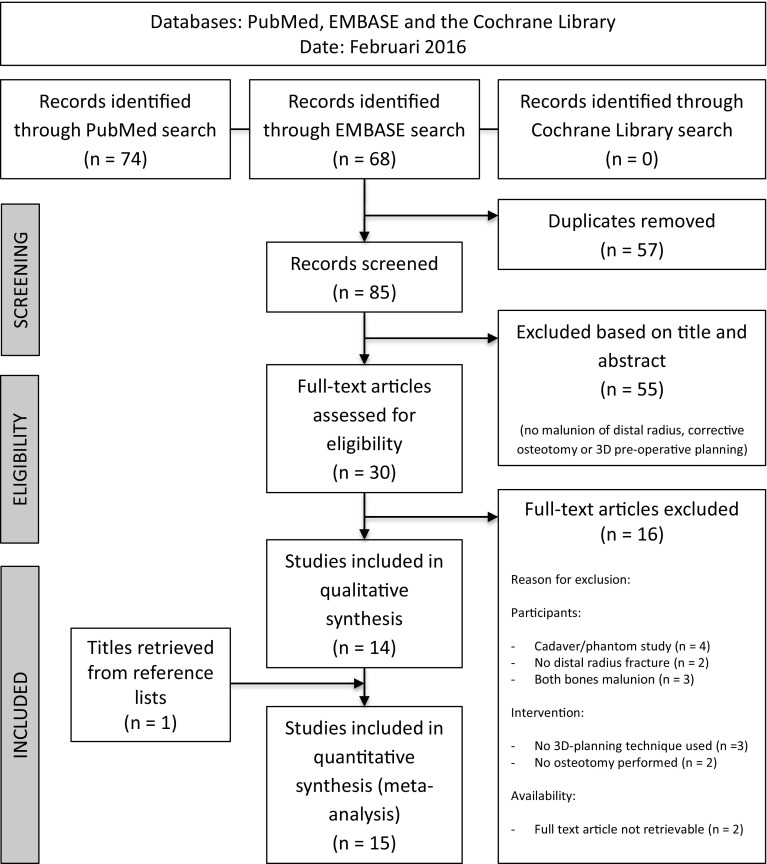
PRISMA flowchart of literature search

The results of the literature search are summarized in a flowchart (Fig. 1). Quality assessment of included studies is summarized in Table 4 and “Appendix” section.

**Table 4:**
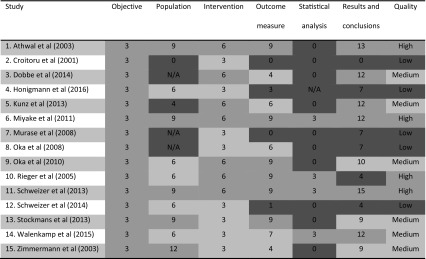
Results of critical appraisal (color figure online)

### Included studies

 Fifteen studies involving 68 participants met the inclusion criteria. Study characteristics are shown in Table 5. Twelve studies are descriptive case-series studies (therapeutic level IV evidence) with sample sizes ranging from two to eleven participants; the remaining three studies are case report studies (therapeutic level V evidence). Additional data were requested for 11 out of 15 titles and were received from two authors [22, 29]. Another author reported that the requested data were not available.

**Table 5:** Characteristics of the included studies

Study	References	Patients in study (*n*)	Mean age in years (range)	Months between injury and osteotomy (range)	Intraoperative technique used
1	Athwal et al. [19]	6	50 (43–60)	9.3 (5–17)	Intraoperative guidance system
2	Croitoru et al. [28]	6	N/A	N/A	Intraoperative guidance system
3	Dobbe et al. [30]	1	40	360	Patient-specific surgical guide and plate
4	Honigmann et al. [29]	1	54	13	Patient-specific surgical guide
5	Kunz et al. [26]	1	61	8	Patient-specific surgical guide
6	Miyake et al. [21]	10	56 (27–79)	48 (2–360)	Patient-specific surgical guide
7	Murase et al.[22]	8	49 (19–72)	12 (5–23)	Patient-specific surgical guide
8	Oka et al. [31]	1	32	5	Patient-specific surgical guide
9	Oka et al. [16]	2	33 (18–48)	6 (4–8)	Patient-specific surgical guide, 3D-cut bone graft
10	Rieger et al. [23]	11	N/A	N/A	Manufactured repositioning device
11	Schweizer et al. [32]	6	48 (33–63)	9 (3–16)	Patient-specific surgical guide
12	Schweizer et al. [25]	2	32 (15–62)	21 (4–48)	Patient-specific surgical guide
13	Stockmans et al. [33]	4	54 (28–66)	9 (6–16)	Patient-specific surgical guide
14	Walenkamp et al. [27]	3	46 (18–64)	31 (14–61)	Visualization, patient-specific surgical guide
15	Zimmermann et al. [34]	6	26 (19–32)	12 (6–14)	Patient-specific surgical guide
Available for IPD (*n*)		–	46	39	–
Mean (SD)		–	51 (SD 17)	30 (SD 79)	–

*N/A* not applicable, *IPD* individual patient data, *SD* standard deviation

### Participants

Of 68 included participants, 16 (23.5%) were men, 28 (41.2%) were woman; for 24 (35.3%) patients gender was not specified. Mean age of the participants was 51 (SD 17, range 15–79) years at the time of surgery. All participants suffered from a symptomatic, malunited fracture of the distal radius. For 25 participants, the initial fracture type was not specified [16, 23, 28, 30]; the remaining fractures were extra-articular (*n* = 28) or combined extra- and intra-articular (*n* = 15) in nature. Initial treatment comprised plaster cast immobilization with or without closed reduction in 34 patients and open reduction and internal fixation in 7 patients. Four studies did not report the initial treatment (*n* = 27) [16, 22, 23, 28]. The mean time between injury and the corrective osteotomy was specified for 38 patients and was 30 months (SD 80, range 2–360).

### Preoperative work up

In all studies, computed axial tomography (CAT scan) was performed to plan the corrective osteotomy: All scans were bilateral except of two cases that focused solely on the correction of an intra-articular step-off. CAT data were used to create a 3D surface mesh of the scanned bones: The affected limb was then superimposed on a mirrored version of the healthy contralateral side. All studies used dedicated software to simulate a rotational, opening or closing wedge osteotomy and to virtually realign the bones.

### Transfer of preoperative plan to patient

The majority of studies (10 out of 15) relied on the use of a custom-made osteotomy template with guiding holes and an osteotomy slit [16, 21, 22, 25–27, 29–33]. Athwal et al. [19] and Croitoru et al. [28] used an optical tracking device to guide the position of drill and screws. Three studies performed the osteotomy by hand but relied on a custom-made wedge-shaped repositioning device that was interposed in the osteotomy gap either temporarily [23, 29] or permanently [16].

With regard to fixation method, volar or dorsal plating with standard implants was the preferred method in the majority of studies. Five studies used a digitalized model of a standardized fixation plate to plan its exact position intra-operatively. Dobbe et al. [30] created a patient-specific plate, which fitted the geometry of the patient’s bone in the realigned position.

### Functional results

Functional outcomes are depicted in Table 6. Mean flexion–extension, pro-supination and grip strength showed statistically significant improvement (*p* < 0.05).

**Table 6:** Functional results of the included studies

Study	ROM wrist		ROM forearm		Grip strength		Complications
	Flexion/extension (°)		Pro-/supination (°)				
	PREOP	POSTOP	PREOP	POSTOP	PREOP	POSTOP	
1	N/A	47/42	N/A	78%/74%^a^	N/A	30 kg, 79% of healthy side	1 partial laceration of EPL tendon. 1 implant removal
2	N/A	N/A	N/A	N/A	N/A	N/A	N/A
3	10/30	60/60	45/45	60/70	Intact	N/A	N/A
4	70/40	70/70	70/40	70/80	N/A	N/A	N/A
5	N/A	N/A	N/A	N/A	N/A	N/A	N/A
6	33/63	63/67	71/76	81/84	39% of healthy side	82% of healthy side	2 postop. Screw loosening^d^. 1 implant removal for EPL tendon problem
7	33/54	62/66	58/69	79/78	42% of healthy side	86% of healthy side	1 distal radioulnar subluxation persisted. 3 implant removal
8	5/45	70/80	N/A	N/A	42% of healthy side	86% of healthy side	N/A
9	83^b^	113^b^	120^c^	150^c^	N/A	N/A	1 implant removal
10	63/59	76/75	50/53	53/65	N/A	N/A	N/A
11	37/49	56/62	69/55	78/80	N/A	Improved with 10%	N/A
12	30/60	50/60	60/80	80/80	N/A	N/A	N/A
13	N/A	N/A	N/A	N/A	N/A	N/A	N/A
14	153^b^	153^b^	165^c^	175^c^	N/A	97% of healthy side	1 distal radioulnar subluxation persisted
15	N/A	N/A	N/A	N/A	N/A	N/A	N/A
Available for IPD (n)	32	39	30	37	23	32	–
Mean (SD)	91 (SD 34)	123 (SD 29)	132 (SD 36)	159 (SD 21)	47% (SD 25) of healthy side	84% (SD 14) of healthy side	–
Pre-postop difference	*p* < 0.05		*p* < 0.05		*p* < 0.05		

*ROM* range of motion, *N/A* not applicable, *PREOP* preoperative, *POSTOP* postoperative, *SD* standard deviation, *EPL* extensor pollicis longus

^a^Range of motion of the forearm is measured as global percentage value

^b^Range of motion of the wrist was measured as the total flexion–extension angle

^c^Range of motion of the forearm was measured as the total rotational arc of the forearm

^d^Both patients with early postoperative screw loosening had osteoporosis

### Radiographic results

Radiographic results are found in Tables 7 and 8. Radiographic evaluation was based on plain radiography (true anteroposterior and lateral views, *n* = 29) or on postoperative CAT scan of the radius (*n* = 19). In addition to CAT evaluation, 14 patients were evaluated by comparing the same 3D planning techniques that were used for the planning of the procedure [27, 30, 32, 33]. Improvement on palmar tilt, radial inclination and ulnar variance showed statistical significance (*p* < 0.05). In all but three cases, directions were improved to within 5° of their normal value. Mean intra-articular step-off improved statistically significant to 0.9 mm. Intra-articular gap was specified in 4 patients only and did not improve significantly.

**Table 7:** Radiographic results of the included studies

Study	Mean time to bone union (weeks, range)	Palmar tilt (°)				Radial inclination (°)		Ulnar variance (mm)	
		PREOP		POSTOP		PREOP	POSTOP	PREOP	POSTOP
		Palmar	Dorsal	Palmar	Dorsal				
1	10.5 (9–12)	21	30	9	–	12	21	7.5	1.9
2	N/A	N/A		N/A		N/A		N/A	
3	N/A	N/A		N/A		N/A		N/A	
4	6	35		9		25	25	5	−1
5	N/A	39	–	4	–	22	26	5	−2
6	16 (8–20)	–	27	13	–	13	24	6	1
7	9.6 (8–12)	–	17	8	–	14	23	3.4	0.6
8	12	N/A		N/A		N/A		N/A	
9	16 (12–20)	28^a^	–	1^a^	–	12^a^	1^a^	N/A	
10	N/A	26	31	10	–	20	22	5.9	0.6
11	8	N/A		N/A		N/A		N/A	
12	N/A	N/A		N/A		N/A		N/A	
13	N/A	−6^b^				−1^b^		0^b^	
14	N/A	19^a^	16^a^	12^a^	8^a^	13^a^	7^a^	5.4^a^	1.7^a^
15	N/A	–	16	10	–	25	23	5.9	1.3
Available for IPD (*n*=)	28	23		27		23	27	23	27
Mean (SD)^c^	12 (SD 3.9)	30 (SD 13)		5 (SD 4)		10 (SD 7)	3 (SD 3)	4.7 (SD 2.5)	1.3 (SD 1.5)
Pre-postop difference	–	*p* < 0.05				*p* < 0.05		*p* < 0.05	

*Preop* preoperative, *postop* postoperative, *IPD* individual patient data, *SD* standard deviation

^a^Palmar tilt and radial inclination are provided as the difference between operated and non-operated side

^b^Palmar tilt, radial inclination and ulnar variance are provided as difference between planned and postoperative result

^c^Distance to normal value (11° volar tilt, 23° radial inclination and neutral (0 mm) ulnar variance, respectively)

**Table 8:** Radiographic results of intra-articular fractures

Study	Intra-articular step-off (mm)		Intra-articular gap (mm)	
	PREOP	POSTOP	PREOP	POSTOP
1	N/A	N/A	N/A	N/A
5	N/A	N/A	N/A	N/A
8	3.0	0.0	N/A	N/A
11	2.7	0.7	N/A	0.0
12	N/A	N/A	N/A	N/A
13	1.1	0.7	2.3	1.4
Available for IPD (*n*=)	11	11	4	4
Mean (SD)	2.5 (SD 0.7)	0.9 (SD 0.6)	2.6 (SD 0.9)	2.1 (SD 0.9)
Pre-postop difference^a^	*p* < 0.05		*p* = 0.72	

*Preop* preoperative, *postop* postoperative, *IPD* individual patient data, *SD* standard deviation

^a^Wilcoxon signed rank test

### Complications

Complications were reported in eleven patients (16%); in two patients, early postoperative screw loosening occurred. These patients required revision surgery with longer plates. One patient suffered from a partial laceration of the extensor pollicis longus tendon, and in two patients distal radioulnar subluxation persisted after surgery. Additionally, six patients had their hardware removed due to hardware-related pain or discomfort. No other complications were observed.

## Discussion

We found that a 3D-planned corrective osteotomy significantly improves both radiographic and functional outcome in patients with a malunited fracture of the distal radius. All included studies reported improvement on radiographic and/or functional parameters with a considerable number of complications.

Unfortunately, our study has not identified studies comparing the results of 3D planning techniques with more conventional planning methods. Moreover, 3D-planning techniques might be reserved for the more complex cases, making it difficult to truly compare cohorts. Nonetheless, some studies show that in conventional osteotomies only 40% of the corrections reach within 5° of the planned correction of the angular deformity (palmar tilt, radial inclination) and within 2 mm of the planned ulnar variance [39]. Other studies report relatively good results of conventional techniques, with significantly improved function for both intra- and extra-articular malunions [40, 41].

Moreover, it is likely that some fractures benefit more from 3D-planned procedures than other. Rotational deformities for instance are difficult to assess and address with conventional planning and are correlated with clinical outcome [18]. Additionally, intra-articular malunions can benefit specifically from a 3D-planned procedure. Articular malunions often require a multiplanar osteotomy, which can be very difficult to perform with conventional planning. 3D planning with patient-specific drill and saw guides can really facilitate this challenging procedure.

Most authors highlight the importance of 3D-planned corrective osteotomies with the fact that 3D-deformations are often not addressed with conventional 2D planning techniques. Vroemen and colleagues have shown that clinical outcome correlates with 3D rotational deficits but not with 2D evaluation parameters [18]. Subsequently, it is remarkable that the majority of studies in this review used conventional radiographs to evaluate the postoperative positioning of the radius instead of an imaging modality that facilitates 3D evaluation. Residual deformities could have been underappreciated, which may have had an influence on the results.

In this systematic review and meta-analysis with the largest cohort yet, we critically appraised available studies focusing on the results of 3D-planned corrective osteotomies of distal radius and performed individual patient data analyses. However, this study is limited by the fact that all included studies had a descriptive character, which makes them highly susceptible to bias. Additionally, a great heterogeneity was seen in type of malunions treated and the technique used for the corrective procedure. Despite this heterogeneity, we chose to combine all patients in one cohort. Due to the diversity of outcome measures, we were forced to transpose data into simplified forms, often losing details in the process. For instance, due to a lack of radiographic data on contralateral extremities, we described radiographic parameters as their distance to a widely accepted normal value. Although we feel this is a valid method with the constraint of limited data availability, this method does not take into account one of the cornerstones of 3D planning techniques.

Disadvantages of the 3D-planning technique include the need for specialized software, the time and effort needed for the preoperative planning, radiation exposure and the costs for the custom-made template and CAT scan. Unfortunately, this review could not shed light on these important aspects of this technique, as data were not provided by any of the included studies. In this systematic review, we found a considerable complication rate of 16%. Corrective osteotomies, however, tend to show higher percentages of complications and do not compare to less complex elective wrist surgery [42].

To fully comprehend the added value of 3D planning corrective osteotomies, we feel a randomized controlled study is inevitable. Leong and colleagues published a protocol for such a trial in 2010, of which the first results are expected early 2018 [43].

With the current advances in 3D printing technology, most techniques reviewed in this study become commercially available. Several companies (e.g., Xilloc BV, Maastricht, The Netherlands or Materialise NV, Leuven, Belgium) provide services to develop patient-specific cutting guides based on CAT data and input by the treating surgeon. The complete process of virtual planning and production of patient-specific implants take 6–8 weeks depending on the complexity of the malunion. Individualized cutting and drilling guides that fit the patients’ bone geometry could make less readily available techniques such as optical tracking devices obsolete. With the importance of accuracy in mind, it is very likely that future osteotomies will go hand in hand with 3D planning techniques.

## Conclusion

3D-planned corrective osteotomies show significant improvement to both functional and radiographic results in patients with a malunion of the distal radius. With the current advances in 3D printing technology, it seems a promising technique in the treatment of complex malunions of the distal radius. However, further research is required to draw a definite conclusion on the added value of 3D-planning techniques.

## Appendix: Critical appraisal scored for included studies

See Table 9.

**Table 9:** Scores for critical appraisal

References	Objective	Population	Intervention	Outcome measure	Statistical analysis	Results and conclusions
	Hypothesis/aim/objective stated clearly?	2. Participants described? 3. Cases collected in more than one center? 4. Eligibility criteria explicit and appropriate? 5. Participants recruited consecutively? 6. Participants entered study at similar point?	7. Intervention clearly described? 8. Additional interventions clearly reported?	9. Outcome measures clearly defined? 10. Relevant outcomes appropriately measured with objective methods? 11. Outcomes measured before and after intervention?	12. Statistical tests used to asses relevant outcomes?	13. Length of follow-up reported? 14. Does the study provide estimates of random variability of relevant outcomes? 15. Adverse events reported? 16. Conclusions supported by results? 17. Competing interests and sourced of support reported?
Athwal et al. [19]	1. 3	2. 3 3. 0 4. 3 5. 0 6. 3	7. 3 8. 3	9. 3 10. 3 11. 3	12. 0	13. 3 14. 0 15. 3 16. 3 17. 3
Croitoru et al. [28]	1. 3	2. 0 3. 0 4. 0 5. 0 6. 0	7. 3 8. 0	9. 0 10. 0 11. 0	12. 0	13. 0 14. 0 15. 0 16. 0 17. 0
Dobbe et al. [30]	1. 3	2. 3 3. Not applicable 4. Not applicable 5. Not applicable 6. Not applicable	7. 3 8. 3	9. 0 10. 1 11. 3	12. 0	13. 3 14. Not applicable 15. 3 16. 3 17. 3
Honigmann et al. [29]	1.3	2. 3 3. 0 4. 3 5. Not applicable 6. Not applicable	7. 3 8. 0	9. 1 10. 1 11. 1	12. N/A	13. 3 14. 0 15. 3 16. 1 17. 0
Kunz et al. [26]	1. 3	2. 1 3. 0 4. 0 5. 0 6. 3	7. 3 8. 3	9. 0 10. 3 11. 3	12. 0	13. 3 14. 0 15. 3 16. 3 17. 3
Miyake et al. [21]	1. 3	2. 3 3. 0 4. 3 5. 0 6. 3	7. 3 8. 3	9. 3 10. 3 11. 3	12. 3	13. 3 14. 0 15. 3 16. 3 17. 3
Murase et al. [22]	1. 3	2. 3 3. Not applicable 4. Not applicable 5. Not applicable 6. Not applicable	7. 3 8. Not applicable	9. 0 10. 0 11. 0	12. 0	13. 3 14. Not applicable 15. 3 16. 1 17. 0
Oka et al. [31]	1. 3	2. 3 3. Not applicable 4. Not applicable 5. Not applicable 6. Not applicable	7. 3 8. Not applicable	9. 3 10. 0 11. 3	12. 0	13. 3 14. Not applicable 15. 3 16. 1 17. 0
Oka et al. [16]	1. 3	2. 3 3. 0 4. 0 5. 0 6. 3	7. 3 8. 3	9. 3 10. 3 11. 3	12. 0	13. 3 14. 0 15. 3 16. 3 17. 1
Rieger et al.[23]	1. 3	2. 0 3. 0 4. 3 5. 0 6. 3	7. 3 8. 3	9. 3 10. 3 11. 3	12. 3	13. 3 14. 0 15. 0 16. 1 17. 0
Schweizer et al. [32]	1. 3	2. 3 3. 0 4. 3 5. 0 6. 3	7. 3 8. 3	9. 3 10. 3 11. 3	12. 3	13. 3 14. 3 15. 3 16. 3 17. 3
Schweizer et al. [25]	1. 3	2. 3 3. 0 4. 0 5. 0 6. 3	7. 3 8. 0	9. 0 10. 0 11. 1	12. 0	13. 3 14. 0 15. 0 16. 1 17. 0
Stockmans et al. [33]	1. 3	2. 3 3. 0 4. 3 5. 0 6. 3	7. 3 8. 0	9. 3 10. 3 11. 3	12. 0	13. 0 14. 3 15. 0 16. 3 17. 3
Zimmermann et al. [34]	1. 3	2. 3 3. 0 4. 3 5. 3 6. 3	7. 3 8. 0	9. 0 10. 1 11. 3	12. 0	13. 3 14. 0 15. 3 16. 3 17. 0
Walenkamp et al. [27]	1.3	2. 3 3. 0 4. 3 5. 0 6. 0	7. 3 8. 0	9. 3 10. 3 11. 1	12. 3	13. 3 14. 0 15. 3 16. 3 17. 3
